# Supplementary material for: Wnt signaling mediates acquisition of blood–brain barrier properties in naïve endothelium derived from human pluripotent stem cells
Source: eLife. 2021 Nov 10;10:e70992. doi: 10.7554/eLife.70992 (PMC8664294; doi:10.7554/eLife.70992)
Supplement: Transparent reporting form [file elife-70992-transrepform1.docx]

***eLife’s* transparent reporting form**

We encourage authors to provide detailed information *within their submission* to facilitate the interpretation and replication of experiments. Authors can upload supporting documentation to indicate the use of appropriate reporting guidelines for health-related research (see [EQUATOR Network](http://www.equator-network.org/%20)), life science research (see the [BioSharing Information Resource](https://biosharing.org/)), or the [ARRIVE guidelines](http://www.plosbiology.org/article/info:doi/10.1371/journal.pbio.1000412) for reporting work involving animal research. Where applicable, authors should refer to any relevant reporting standards documents in this form.

If you have any questions, please consult our Journal Policies and/or contact us: [editorial@elifesciences.org](mailto:editorial@elifesciences.org).

**Sample-size estimation**

- You should state whether an appropriate sample size was computed when the study was being designed
- You should state the statistical method of sample size computation and any required assumptions
- If no explicit power analysis was used, you should describe how you decided what sample (replicate) size (number) to use

Please outline where this information can be found within the submission (e.g., sections or figure legends), or explain why this information doesn’t apply to your submission:

No formal power analysis was used to compute sample size. We performed 3–4 biological replicates for each treatment group, which is consistent with similar *in vitro* studies in the literature. For the assays of interest, we typically observe relative standard deviations of 10–20%, and 3–4 biological replicates is sufficient to detect fold-changes of +/- 0.5 under these conditions with 80% power. We repeated most experiments in multiple hPSC differentiations to confirm robustness of observed effects.

**Replicates**

- You should report how often each experiment was performed
- You should include a definition of biological versus technical replication
- The data obtained should be provided and sufficient information should be provided to indicate the number of independent biological and/or technical replicates
- If you encountered any outliers, you should describe how these were handled
- Criteria for exclusion/inclusion of data should be clearly stated
- High-throughput sequence data should be uploaded before submission, with a private link for reviewers provided (these are available from both GEO and ArrayExpress)

Please outline where this information can be found within the submission (e.g., sections or figure legends), or explain why this information doesn’t apply to your submission:

The general replication strategy is outlined in the Statistics section of Materials and Methods, and specific replication details are present in each figure legend. A definition of biological replication (including within-differentiation and across-differentiation replication) is present in the Statistics section of Materials and Methods; technical replication was not used. We did not encounter or test for outliers. No data were excluded. RNA-sequencing data has been submitted to GEO and the accession number and reviewer token are present in the Data availability section.

**Statistical reporting**

- Statistical analysis methods should be described and justified
- Raw data should be presented in figures whenever informative to do so (typically when N per group is less than 10)
- For each experiment, you should identify the statistical tests used, exact values of N, definitions of center, methods of multiple test correction, and dispersion and precision measures (e.g., mean, median, SD, SEM, confidence intervals; and, for the major substantive results, a measure of effect size (e.g., Pearson's r, Cohen's d)
- Report exact p-values wherever possible alongside the summary statistics and 95% confidence intervals. These should be reported for all key questions and not only when the p-value is less than 0.05.

Please outline where this information can be found within the submission (e.g., sections or figure legends), or explain why this information doesn’t apply to your submission:

Each figure legend contains a description of the statistical test(s) used, definitions of the points shown in figures as biological replicates from one or more differentiations, and definition of the bars (mean). Individual data points representing the N biological replicates are shown in figures. Exact P-values (including those > 0.05) are shown for all key comparisons. Some very small P-values derived from Tukey’s HSD and Dunnett’s post-hoc tests are reported as P < 0.0001 due to limitations of the statistical analysis software. Justification for statistical analysis methods is present in the Statistics section of Materials and Methods.

(For large datasets, or papers with a very large number of statistical tests, you may upload a single table file with tests, Ns, etc., with reference to sections in the manuscript.)

**Group allocation**

- Indicate how samples were allocated into experimental groups (in the case of clinical studies, please specify allocation to treatment method); if randomization was used, please also state if restricted randomization was applied
- Indicate if masking was used during group allocation, data collection and/or data analysis

Please outline where this information can be found within the submission (e.g., sections or figure legends), or explain why this information doesn’t apply to your submission:

For each experiment, replicate cell culture wells for the control and experimental group(s) were all derived from a single pool of cells. Masking/blinding was not used as the same researcher performed cell culture, data acquisition, and data analysis.

**Additional data files (“source data”)**

- We encourage you to upload relevant additional data files, such as numerical data that are represented as a graph in a figure, or as a summary table
- Where provided, these should be in the most useful format, and they can be uploaded as “Source data” files linked to a main figure or table
- Include model definition files including the full list of parameters used
- Include code used for data analysis (e.g., R, MatLab)
- Avoid stating that data files are “available upon request”

Please indicate the figures or tables for which source data files have been provided:

For RNA-sequencing data represented by heat maps in Figure 6, Figure 6–figure supplement 2, and Figure 6–figure supplement 5, corresponding numerical data are present in Supplementary file 1. Supplementary files 1, 2, and 3 additionally contain complete transcript abundance and differential expression analysis results. All other figures contain numerical data as individual data points representing the biological replicates.
